# Eliminating paediatric infections and keeping mothers alive

**DOI:** 10.7448/IAS.15.6.18082

**Published:** 2012-11-11

**Authors:** G Gray

**Affiliations:** University of the Witwatersrand, Johannesburg, South Africa

## Abstract

The global plan of reducing the number of new child HIV infections and a reduction in the number of HIV-related maternal deaths by 2015 will require inordinate political commitment and strengthening of health systems in Sub-Saharan Africa where the burden of HIV infections in pregnant women is the highest. Preventing HIV infection in women of child-bearing age and unwanted pregnancies in HIV-positive women forms the cornerstone of long-term control of paediatric HIV infections. To achieve the goal of eliminating paediatric HIV infection by 2015, health systems strengthening to address prevention of mother-to-child HIV transmission cascade attrition and focusing on the elimination of breastmilk transmission is critical. Understanding the pathogenesis of breastmilk transmission and the mechanisms by which antiretroviral therapy impacts on transmission through this compartment will drive future interventions. Identifying and retaining HIV-positive pregnant women in care and committed to long-term antiretroviral therapy will improve maternal outcomes and concomitant reductions in maternal mortality. Research assessing the natural history of HIV infection and long-term outcomes in women who interrupt antiretroviral therapy post-weaning is urgently required. Data on the outcome of women who opt to continue the long-term use of antiretroviral therapy after initiating therapy during pregnancy will determine future policy in countries considering option B+. The prevalence of antiretroviral resistance and impact on survival in infants who sero-convert whilst receiving neonatal prophylaxis, or are exposed to maternal HAART through breastmilk at a population level, are currently unknown. In addition to the provision of biomedical interventions, healthcare workers and policy makers must address the structural, cultural and community issues that impact on treatment uptake, adherence to medication and retention in care.

